# Supplementary material for: Counterfactual analysis: Comparing voucher systems with taxes, subsidies, and public spending to reduce income inequality
Source: F1000Res. 2026 Jan 30;14:439. Originally published 2025 Apr 15. [Version 3] doi: 10.12688/f1000research.162864.3 (PMC12972710; doi:10.12688/f1000research.162864.3)
Supplement: Peer review history

# Reviewer report: Reviewer response for version 3

**DOI:** 10.5256/f1000research.194945.r460196

**Published:** 2026-03-09

**Authors:** Quoc Huy Nguyen

**Affiliations:** 1 Faculty of Finance and Accounting, Lac Hong University, Biên Hòa, Dong Nai, Vietnam

**- Fatal Flaw in the Econometric Strategy (The Voucher Variable):** The most alarming issue lies in the creation and use of the voucher variable. The authors explicitly state that vouchers are not directly measured in their dataset. Instead, they construct a counterfactual scenario by allocating an average of 0.5% to 1% of GDP to vouchers. They then insert this hypothetical variable into a fixed-effects panel data equation. Furthermore, the manuscript states, "Suppose we obtain estimated coefficients... from our simulation". In empirical econometrics, one cannot regress historical panel data on a non-existent, hypothetical variable, nor can one "suppose" regression coefficients to draw empirical conclusions. The counterfactual analysis is fundamentally invalid as currently constructed.

**- Unresolved Endogeneity:** The manuscript notes that "Tax Revenue was suspected to be endogenous" and references a previous instrumental variable (IV) correction. However, the current model Abandons the IV approach, relying instead on cluster-robust standard errors. Cluster-robust standard errors correct for heteroscedasticity and serial correlation; they do not resolve endogeneity or reverse causality bias.

**- Theoretical Conflation of Revenue and Expenditure:** Echoing previous reviewer feedback, the theoretical framework lacks a clear distinction between the supply-side mechanisms of revenue generation (progressive taxation) and the demand-side mechanisms of redistribution (vouchers and subsidies). These are distinct fiscal tools that operate through different economic channels and should not be conflated in the theoretical buildup.

**Drafting and Presentation Flaws**

 The manuscript currently contains text that reads like an author's drafting notes or a textbook explanation rather than a finalized academic report. For instance, the authors write: "For example, in our results table, we might note that the model explains, say, 50% of the within-country variation in the Gini index". This is unacceptable for a finalized peer-reviewed article.

Is the work clearly and accurately presented and does it cite the current literature?

Partly

If applicable, is the statistical analysis and its interpretation appropriate?

No

Are all the source data underlying the results available to ensure full reproducibility?

No

Is the study design appropriate and is the work technically sound?

Partly

Are the conclusions drawn adequately supported by the results?

No

Are sufficient details of methods and analysis provided to allow replication by others?

No

Reviewer Expertise:

Financial Economics; Development Economics; Banking and Finance.

I confirm that I have read this submission and believe that I have an appropriate level of expertise to confirm that it is of an acceptable scientific standard, however I have significant reservations, as outlined above.

# Reviewer report: Reviewer response for version 3

**DOI:** 10.5256/f1000research.194945.r457460

**Published:** 2026-02-25

**Authors:** Kaepae Ken Ail

**Affiliations:** 1 Papua New Guinea (PNG) University of Technology, Lae, Papua New Guinea

This study is focused on governments' redistribution to critical public sectors (education and health) and not from the supply side (how revenues are raised to finance the public sector). This article focuses on  the voucher systems and subsidies as modes of redistribution to the education and health sectors. These have diverse delivery modes whose effectiveness are different in high, middle and low income nations assessed. However, the article is not clear and mix-up with the taxation aspect. I understand that progressive taxation is a means to raise the revenue required for public use (from taxing labour, consumption and corporate profits)  through redistribution modes such as the voucher systems and subsidies. Also, the progressive taxation has an indirect  redistribution effect on poverty reduction through progressive labour income and household and supply side productivity. Thus, there is no content on tax progressiveness and how it plays a part in redistribution to reduce poverty. The content of the article is excellent, and the findings are important for policy application. However, I recommend changing the title by removing "taxes and tax progressivity" as these are not adequately covered in the entire literature review, methodology and not directly connected anywhere in the results and the interpretation.   

Is the work clearly and accurately presented and does it cite the current literature?

Partly

If applicable, is the statistical analysis and its interpretation appropriate?

Yes

Are all the source data underlying the results available to ensure full reproducibility?

Yes

Is the study design appropriate and is the work technically sound?

Yes

Are the conclusions drawn adequately supported by the results?

Yes

Are sufficient details of methods and analysis provided to allow replication by others?

Yes

Reviewer Expertise:

Reviewer is a prominent academic and consultant in Mining Engineering and Mineral Economics, currently serving as a lecturer at the Papua New Guinea University of Technology (PNGUoT). He is a specialist in mineral taxation,  fiscal policies and economic evaluation of resource projects.

I confirm that I have read this submission and believe that I have an appropriate level of expertise to confirm that it is of an acceptable scientific standard, however I have significant reservations, as outlined above.

## References

[1] : Progressive Taxation.2023; 10.1007/978-3-031-17299-1_2285 5503-5506 10.1007/978-3-031-17299-1_2285

[2] : Estimating the effect of Progressive Personal Income Tax Schedule for Russia in a global CGE-OLG model. *Journal of Tax Reform* .2024;10(2) : 10.15826/jtr.2024.10.2.172 334-354 10.15826/jtr.2024.10.2.172

[3] : Progressive Taxation for Extractive Industries: Summary of a Thesis on Reconciling the Progressive Mineral Taxation Regime of Papua New Guinea. *SSRN Electronic Journal* .2024; 10.2139/ssrn.4943820 10.2139/ssrn.4943820

[4] : A Critical Review of Book: The Taxation of Mineral Rent by Garnaut Ross and Clunnies Ross, 1983. *SSRN Electronic Journal* .2025; 10.2139/ssrn.5115432 10.2139/ssrn.5115432

# Reviewer report: Reviewer response for version 2

**DOI:** 10.5256/f1000research.185196.r412510

**Published:** 2025-09-25

**Authors:** Abdulrasheed Zakari

**Affiliations:** 1 University of Wollongong, Wollongong, New South Wales, Australia

The paper titled *“Contrafactual Analysis: Comparing Voucher Systems with Taxes, Subsidies, and Public Spending to Reduce Income Inequality”* presents an interesting topic and offers some elements of novelty. However, several areas require significant improvement before it can be considered for publication.

**General Comments:**

1. The reporting format of the study diverges from the conventional structure expected in academic articles, making it less accessible to readers.
2. The overall readability of the manuscript needs substantial improvement to ensure clarity and coherence.

**Specific Comments:**

1. The paper lacks a clear theoretical framework linking the key variables, particularly the connection between voucher programs and income inequality.
2. There is no evidence of pre-estimation analysis to demonstrate the appropriateness of the data and variables. Given the use of panel data, it is important to conduct tests such as cross-sectional dependence, multicollinearity, and unit-root tests.
3. The results are not sufficiently aligned or contrasted with findings from previous studies, which weakens the contribution to existing literature.
4. The analysis does not include robustness checks, which are necessary to validate the reliability of the results.

Is the work clearly and accurately presented and does it cite the current literature?

Yes

If applicable, is the statistical analysis and its interpretation appropriate?

Partly

Are all the source data underlying the results available to ensure full reproducibility?

Yes

Is the study design appropriate and is the work technically sound?

Partly

Are the conclusions drawn adequately supported by the results?

Partly

Are sufficient details of methods and analysis provided to allow replication by others?

Partly

Reviewer Expertise:

applied econometric

I confirm that I have read this submission and believe that I have an appropriate level of expertise to state that I do not consider it to be of an acceptable scientific standard, for reasons outlined above.

# Response

**Published:** 2025-11-05

**Authors:** William Pacheco

**Affiliations:** Economics, University Anahuac Mexico, Huixquilucan de Degollado, State of Mexico, Mexico

We sincerely thank the reviewer for taking the time to assess our manuscript and for providing thoughtful feedback. We respectfully acknowledge the reviewer’s conclusion regarding the scientific standard of the paper in its previous form. We have carefully considered each of the points raised and have substantially revised the manuscript to address all identified issues. The new version (Version 3) includes a clearer theoretical framework, detailed pre-estimation diagnostics, enhanced discussion aligned with recent literature, and additional robustness checks to strengthen the validity and reliability of the results. We hope that these comprehensive revisions now meet the journal’s scientific standards and expectations.

# Response

**Published:** 2026-01-07

**Authors:** William Pacheco

**Affiliations:** Economics, University Anahuac Mexico, Huixquilucan de Degollado, State of Mexico, Mexico

We sincerely thank the reviewer for their constructive and detailed feedback. All requested revisions have been carefully addressed: the paper now follows a standard academic structure, includes a strengthened theoretical framework linking voucher programs and income inequality, and incorporates full pre-estimation diagnostics (Pesaran CD, VIF, and LLC/IPS tests). The results section has been expanded with clearer discussion, literature alignment, and robustness checks, while the counterfactual scenarios have been refined to illustrate policy implications more effectively. We believe these revisions have significantly improved the clarity, methodological rigour, and overall contribution of the manuscript.

# Reviewer report: Reviewer response for version 2

**DOI:** 10.5256/f1000research.185196.r407678

**Published:** 2025-09-10

**Authors:** Małgorzata Szczepaniak

**Affiliations:** 1 Nicolaus Copernicus University in Torun, Torun, Poland

In my assessment, the authors have made a considerable effort to incorporate the suggested revisions and have substantially enhanced the scientific quality of the manuscript. I consider the article, in its current form, ready for acceptance for indexing.

Is the work clearly and accurately presented and does it cite the current literature?

Partly

If applicable, is the statistical analysis and its interpretation appropriate?

Partly

Are all the source data underlying the results available to ensure full reproducibility?

Partly

Is the study design appropriate and is the work technically sound?

Partly

Are the conclusions drawn adequately supported by the results?

Yes

Are sufficient details of methods and analysis provided to allow replication by others?

Yes

Reviewer Expertise:

Economics; income inequalities; economic stratification; life satisfaction

I confirm that I have read this submission and believe that I have an appropriate level of expertise to confirm that it is of an acceptable scientific standard.

# Reviewer report: Reviewer response for version 1

**DOI:** 10.5256/f1000research.179131.r381763

**Published:** 2025-06-20

**Authors:** Małgorzata Szczepaniak

**Affiliations:** 1 Nicolaus Copernicus University in Torun, Torun, Poland

Summary of the Article

 This study investigates the relative effectiveness of voucher systems compared to taxes, subsidies, and public spending in reducing income inequality. Through econometric modeling and contrafactual analysis using panel data from 12 countries over 22 years, the authors assess multiple “what-if” scenarios and simulate the effects on the Gini index. They conclude that vouchers—especially when tax-funded—can enhance redistribution, particularly in essential services like health and education.

 Even though my overall evaluation of the article is good, particularly because of the innovative use of a contrafactual simulation and policy implications, I would like to suggest some improvements that are recommended to introduce to increase the quality and transparency of the text.

1. Clarity and Literature. Some conceptual transitions between the theoretical background and the model implementation (especially regarding vouchers) lack precision. Recent empirical evaluations of large-scale voucher programs (e.g., Chile’s school voucher reforms, healthcare vouchers in Thailand) could strengthen the literature base. Some core citations in this area are missing, i.e.: Andreas Bergh. (2005) (Ref 1);  Doerrenberg, Philipp and Peichl, Andreas, (2012) (Ref 2)
2. If the empirical part doesn't concern UBI, it is not worth discussing so broadly in the theoretical background, but it would be rather suggested to delve deeper into the part and description of the introduction of voucher systems, i.e., real cases.
3. Design and Technical Soundness The voucher variable is introduced as a simulated construct rather than an empirically observed variable. There is little validation offered for the assumptions behind this simulation. The model does not account for time-lag effects or program maturity, which could be important for redistributive outcomes
4. Statistical Appropriateness The statistical analysis is mostly correct, but: (1) There is no model fit information (e.g., R² or within-between variance); (2) No robustness checks; (3) No statistical significance for correlation matrix is presented; moreover, the presentation of correlation matrix should be unified.

Is the work clearly and accurately presented and does it cite the current literature?

Partly

If applicable, is the statistical analysis and its interpretation appropriate?

Partly

Are all the source data underlying the results available to ensure full reproducibility?

Partly

Is the study design appropriate and is the work technically sound?

Partly

Are the conclusions drawn adequately supported by the results?

Yes

Are sufficient details of methods and analysis provided to allow replication by others?

Yes

Reviewer Expertise:

Economics; income inequalities; economic stratification; life satisfaction

I confirm that I have read this submission and believe that I have an appropriate level of expertise to confirm that it is of an acceptable scientific standard, however I have significant reservations, as outlined above.

## References

[1] : On the Counterfactual Problem of Welfare State Research: How Can We Measure Redistribution?. *European Sociological Review* .2005;21(4) : 10.1093/esr/jci024 345-357 10.1093/esr/jci024

[2] : The impact of redistributive policies on inequality in OECD countries. *Applied Economics* .2014;46(17) : 10.1080/00036846.2014.892202 2066-2086 10.1080/00036846.2014.892202

# Response

**Published:** 2025-07-15

**Authors:** William Pacheco

**Affiliations:** Economics, University Anahuac Mexico, Huixquilucan de Degollado, State of Mexico, Mexico

Thank you very much for your thoughtful and constructive review. I appreciate the detailed feedback and suggestions, which are extremely helpful in improving the clarity and rigour of the manuscript. I will incorporate the recommended changes, particularly in strengthening the theoretical framework, integrating recent empirical studies, validating the simulated voucher variable, and enhancing the statistical reporting. I appreciate your time and insights.
